# Structural brain changes in patients with post-COVID fatigue: a prospective observational study

**DOI:** 10.1016/j.eclinm.2023.101874

**Published:** 2023-02-27

**Authors:** Josephine Heine, Katia Schwichtenberg, Tim J. Hartung, Sophia Rekers, Claudia Chien, Fabian Boesl, Rebekka Rust, Christian Hohenfeld, Julia Bungenberg, Ana S. Costa, Carmen Scheibenbogen, Judith Bellmann-Strobl, Friedemann Paul, Christiana Franke, Kathrin Reetz, Carsten Finke

**Affiliations:** a Department of Neurology, Charité – Universitätsmedizin Berlin, Corporate Member of Freie Universität Berlin and Humboldt-Universität zu Berlin, Berlin, Germany; b Berlin School of Mind and Brain, Humboldt-Universität zu Berlin, Berlin, Germany; c Experimental and Clinical Research Center, Charité - Universitätsmedizin Berlin Corporate Member of Freie Universität Berlin, Humboldt-Universität zu Berlin, and Berlin Institute of Health and Max Delbrück Center for Molecular Medicine, Berlin, Germany; d NeuroCure Clinical Research Center (NCRC), Charité – Universitätsmedizin Berlin, Freie Universität Berlin, Humboldt Universität zu Berlin, Berlin Institute of Health (BIH), Berlin, Germany; e Department of Neurology, RWTH Aachen University, Aachen, Germany; f JARA Brain Institute Molecular Neuroscience and Neuroimaging (INM-11), Research Centre Jülich and RWTH Aachen University, Aachen, Germany; g Institute for Medical Immunology, Charité – Universitätsmedizin Berlin, Corporate Member of Freie Universität Berlin and Humboldt Universität zu Berlin, Berlin, Germany; h Berlin Center for Advanced Neuroimaging, Charité – Universitätsmedizin Berlin, Corporate Member of Freie Universität Berlin and Humboldt-Universität zu Berlin, Berlin, Germany

**Keywords:** Post-COVID syndrome, Fatigue, COVID-19, Neuroimaging, Basal ganglia, Thalamus

## Abstract

**Background:**

Post-COVID syndrome is a severe long-term complication of COVID-19. Although fatigue and cognitive complaints are the most prominent symptoms, it is unclear whether they have structural correlates in the brain. We therefore explored the clinical characteristics of post-COVID fatigue, describe associated structural imaging changes, and determine what influences fatigue severity.

**Methods:**

We prospectively recruited 50 patients from neurological post-COVID outpatient clinics (age 18–69 years, 39f/8m) and matched non-COVID healthy controls between April 15 and December 31, 2021. Assessments included diffusion and volumetric MR imaging, neuropsychiatric, and cognitive testing. At 7.5 months (median, IQR 6.5–9.2) after the acute SARS-CoV-2 infection, moderate or severe fatigue was identified in 47/50 patients with post-COVID syndrome who were included in the analyses. As a clinical control group, we included 47 matched multiple sclerosis patients with fatigue.

**Findings:**

Our diffusion imaging analyses revealed aberrant fractional anisotropy of the thalamus. Diffusion markers correlated with fatigue severity, such as physical fatigue, fatigue-related impairment in everyday life (Bell score) and daytime sleepiness. Moreover, we observed shape deformations and decreased volumes of the left thalamus, putamen, and pallidum. These overlapped with the more extensive subcortical changes in MS and were associated with impaired short-term memory. While fatigue severity was not related to COVID-19 disease courses (6/47 hospitalised, 2/47 with ICU treatment), post-acute sleep quality and depressiveness emerged as associated factors and were accompanied by increased levels of anxiety and daytime sleepiness.

**Interpretation:**

Characteristic structural imaging changes of the thalamus and basal ganglia underlie the persistent fatigue experienced by patients with post-COVID syndrome. Evidence for pathological changes to these subcortical motor and cognitive hubs provides a key to the understanding of post-COVID fatigue and related neuropsychiatric complications.

**Funding:**

10.13039/501100001659Deutsche Forschungsgemeinschaft (DFG) and German Ministry of Education and Research (BMBF).

**Research in context**

**Evidence before this study**

We searched Pubmed for original articles, meta-analyses, and systematic reviews published until June 15, 2022. Our search terms included “post[-]COVID”/”long[-]COVID”/“COVID[-]19”/”SARS-CoV-2” AND “fatigue”/”neurol∗”/”psychiatr∗” OR “imaging”/”MR∗/”neuronal”/”brain”. We found meta-analyses, observational cohort studies, and larger case series (>100 patients) reporting an estimated prevalence of postacute fatigue of ∼30% patients with COVID-19 that is associated with cognitive deficits and decreased quality of life. A large study using data from the UK Biobank reported brain changes related to SARS-CoV-2 infection, albeit without a specific clinical characterisation regarding fatigue or defining a post-COVID diagnosis. We found no publications on neuroimaging changes in samples of patients with post-COVID fatigue.

**Added value of this study**

To our best knowledge, our prospective observational study provides the first evidence of structural brain changes in patients with moderate or severe fatigue due to post-COVID syndrome. Using MRI-based volumetry and diffusion imaging, we show that the persistent fatigue symptoms in patients with post-COVID syndrome have a distinct neuronal substrate and are associated with fatigue levels, daytime sleepiness, and neuropsychiatric symptoms. These structural changes to major subcortical motor and cognitive hubs provide a basis for the understanding of this long-term consequence of COVID-19.

**Implications of all the available evidence**

By showing that the subjective symptom of fatigue has an underlying structural correlate in the brain, we provide an insight into the brain changes related to post-COVID syndrome and report a potential longitudinal biomarker for recovery. In the context of further increasing numbers of SARS-CoV-2 infections, a precise characterisation of post-COVID fatigue is a prerequisite to understand the involved pathomechanisms and improve patient care.

## Introduction

Post-COVID syndrome (PCS) frequently manifests with neurological symptoms, including fatigue, pain, and headache.^1^^,^^2^ This newly recognised syndrome has been defined as continued or new onset symptoms that persist for a least 3 months after COVID-19 and compromise everyday functioning.^3^ In fact, cognitive complaints and fatigue are among the major neurological symptoms of patients with post-COVID syndrome and severely impact their quality of life.^4^^,^^5^ However, the pathomechanisms and imaging correlates of this novel disease entity still remain elusive.

Fatigue is characterised by an overwhelming experience of weakness, exhaustion, and decreased capacity for physical or mental work which is disproportional to recent activity.^6^, ^7^, ^8^ Fatigue is a complex phenomenon at the interplay of central regulation (i.e., homeostasis) and psychological factors like mood and motivation.^6^^,^^7^^,^^9^ In PCS, the experience of fatigue is frequently accompanied by muscle fatigue and fatiguability, which can be assessed by hand grip strength and correlates with biomarkers for inflammation and hypoperfusion.^10^ A subset of patients with PCS fulfil the diagnostic criteria for myalgic encephalomyelitis/chronic fatigue syndrome (ME/CFS), a complex disease in which fatigue is accompanied by exertional intolerance and post-exertional malaise. Environmental stressors may additionally contribute to fatigue through changes in the neuroendocrine stress response, altering the hypothalamic-pituitary-adrenal and norepinephrine systems.^7^

Central fatigue impacts psychosocial and cognitive functions and is often perceived as most distressing by patients.^7^ In fact, fatigue is one of the main contributors to post-COVID disease burden.^11^ A recent meta-analysis estimated that ∼30% of the patients with COVID-19 develop post-COVID fatigue.^12^ In line with this, we recently found fatigue to be more than twice as common after COVID-19 as in matched non-COVID controls. Importantly, fatigue frequently affected younger patients and patients with only mild or moderate acute COVID-19.^13^^,^^14^ In addition, post-COVID cognitive deficits are observed in up to 20% of patients, especially in older age groups.^12^ These deficits can span multiple domains, including short-term and episodic memory, attention, and language^15^ and they can occur with a delay of several weeks or months after infection.^4^

The prominent neurological symptoms in acute COVID-19 and PCS indicate a direct or indirect involvement of the central nervous system, potentially mediated by direct virus effects or virus-induced autoimmunity.^16^^,^^17^ Indeed, neuropathological and imaging studies revealed neuroinflammatory and vascular injury in severely ill patients with COVID-19.^18^^,^^19^ In previously hospitalised patients who developed PCS, subcortical white matter lesions were reported 4 months after discharge.^15^ In the largest study to date, even patients with mild acute COVID-19 and without hospitalisation showed greater longitudinal reductions in grey matter thickness and reduced global brain volumes compared to healthy controls.^2^

Previous research in neuroimmunological disorders identified robust associations of structural brain alterations with fatigue severity and cognitive impairment. For example, basal ganglia changes^20^^,^^21^ and frontoparietal white matter damage^22^ have consistently been linked to fatigue in multiple sclerosis, while hippocampal damage is associated with memory deficits across diseases.^23^^,^^24^ In the case of post-COVID syndrome, however, it remains currently unclear whether such distinct lesion patterns exist and whether structural brain damage underlies the persisting neurological symptoms in patients with PCS.

Here, we (I) report the characteristics of persistent fatigue in a cohort of patients with post-COVID syndrome; (II) analyse the integrity of white matter and subcortical structures using MR diffusion and volumetric imaging; (III) determine to what extent fatigue-related structural brain changes overlap with those observed in other aetiologies (i.e., multiple sclerosis); and (IV) determine whether features of the acute COVID-19 course predict subsequent post-COVID fatigue and associated structural brain changes.

## Methods

### Study design and participants

#### Post-COVID patients

We prospectively recruited 50 patients from the neurological post-COVID outpatient clinic at Charité – Universitätsmedizin Berlin, Germany, between April 15 and November 30, 2021. Inclusion criteria were (1) a history of confirmed SARS-CoV-2 infection (i.e., positive RT-PCR test) with (2) postinfectious neurological symptoms for at least 3 months and (3) no history of relevant neurological disease prior to COVID-19. The CAMINO (Cognition and MRI in post-COVID) study protocol comprised structural MR imaging, cognitive testing, and an assessment of fatigue and neuropsychiatric outcomes on the day of the study visit. We identified 47 patients (age 43.4 (11.9) years) with moderate or severe fatigue levels based on the cut-offs provided by the Fatigue Scale for Motor and Cognitive Function (FSMC ≥ 53) who were included in this cross-sectional study for further analyses. Clinical details are presented in Table 1.

**Table 1:** Clinical characteristics.

Acute COVID-19 disease course	
Age (years)	43.4 (11.9), range 18–69
Sex	39 f, 8 m
Hospitalisation	13% (6/47)
Intensive care unit (ICU)	4% (2/47)
Oxygen supplementation	17% (8/47)
Ventilation	9% (4/47)
COVID-19 disease duration (days)^a^	median 21 (IQR 21–28)
COVID-19 symptom count^b^	median 3 (IQR 2–4)
Acute COVID-19 symptoms^b^	
Headache	47% (22/47)
Hyposmia/hypogeusia	45% (21/47)
Limb pain	30% (14/47)
Fever	26% (12/47)
Dyspnoea	21% (10/47)
Cough	17% (8/47)
Thoracic pain	17% (8/47)
Abdominal pain	6% (3/47)
Sore throat	15% (7/47)
Shivering	13% (6/47)
Rhinitis	11% (5/47)
Diarrhoea	4% (2/47)

Post-COVID syndrome^e^		
Post-COVID disease duration (months)	median 7.5 (IQR 6.5–9.2)	
Post-COVID mRS	median 2 (IQR 2–2.5)	
ME-CFS criteria (% of patients)^c^	29% (12/41)	*controls*: 0% (0/47)
Beck Anxiety Inventory (BAI)	14.6 (7.5)	*controls*: 3.9 (4.8)
Beck Depression Inventory (BDI-II)	17.0 (7.8)	*controls*: 4.8 (5.4)
Bell score	48.5 (16.7)	*controls*: 97.0 (6.3)
Epworth Sleepiness Scale (ESS)	11.2 (5.6)	*controls*: 5.5 (3.2)
Fatigue Scale for Motor & Cognitive Function (FSMC)	77.0 (10.1)	*controls*: 33.2 (11.1)
Physical fatigue	38.2 (5.6)	*controls*: 16.9 (5.6)
Cognitive fatigue	38.9 (6.1)	*controls*: 16.3 (6.0)
Fatigue Severity Scale (FSS)	55.0 (8.7)	*controls*: 23.1 (10.4)
Pittsburgh Sleep Quality Index (PSQI)	8.2 (3.9)	*controls*: 5.8 (4.1)

Post-COVID neurological symptoms^d^	
Headache	47% (22/47)
Hyposmia/hypogeusia	45% (21/47)
Myalgia	21% (10/47)
Dizziness	11% (5/47)
Vertigo	11% (5/47)
Muscle weakness	9% (4/47)
Sensibility dysfunction	4% (2/47)
Autonomic dysfunction	2% (1/47)
Cerebellar symptoms	2% (1/47)
Movement disorder	2% (1/47)
Extrapyramidal symptoms	2% (1/47)
Oculomotor symptoms	2% (1/47)
Gait	2% (1/47)
Aphasia	2% (1/47)
Apraxia	0% (0/47)
Dysarthria/dysphagia	0% (0/47)
Brain stem symptoms	0% (0/47)
Cerebrovascular incidents	0% (0/47)
Impaired consciousness	0% (0/47)
Paresis	0% (0/47)
Pyramidal tract dysfunction	0% (0/47)
Seizures	0% (0/47)

a Post-COVID syndrome disease duration is defined as the time between the onset of COVID-19 and study assessment.

b Self-reported.

c International consensus criteria for myalgic encephalomyelitis. We limited the 6-months criterion to 3 months due to the novelty of COVID-19 and the recent enrolment of participants.

d Physician-assessed based on medical history and neurological examination (IQR: interquartile range (first and third quartiles), ME-CFS: myalgic encephalomyelitis – chronic fatigue syndrome, mRS: modified Rankin Score.

e Quantitative values are reported with mean (SD) unless otherwise indicated.

#### Healthy controls

Along with the post-COVID patient group, we prospectively recruited a healthy control group without history of neurological or psychiatric diseases and without previous COVID-19 infection. Healthy control participants were individually matched regarding age, sex, and education (n = 47, mean age 44.5 (14.1) years) and received an identical study protocol.

#### MS patients

Moreover, we retrospectively identified a clinical control group of patients with multiple sclerosis and fatigue. Patients with MS were recruited between October 01, 2019 and December 31, 2021 at the NeuroCure Clinical Research Center (NCRC), Charité – Universitätsmedizin Berlin, Germany. Inclusion criteria were (1) a diagnosis of relapsing-remitting multiple sclerosis (RRMS) according to the McDonald 2017 diagnostic criteria and (2) moderate to severe fatigue (Fatigue Severity Scale (FSS) ≥ 1 SD from healthy normative sample).^25^ Patients with MS were individually matched to patients with post-COVID syndrome and healthy controls regarding age and sex (n = 47, mean age 43.1 (10.7) years). Besides increased levels of fatigue (FSS 5.0 (1.0)), the MS group showed mild to moderate MS-related disability (median EDSS 2.5 (IQR 1.5–3.5), *Expanded Disability Status Scale*) at a median of 7.9 years (IQR 4.9–11.3 years) since symptom onset. All participants gave informed written consent. This study was approved by the ethics committee of Charité – Universitätsmedizin Berlin (EA2/007/21) and conducted in accordance with the declaration of Helsinki.

### Imaging data acquisition

Magnetic resonance imaging was performed at the Berlin Centre for Advanced Neuroimaging (BCAN) using a 3T PRISMA scanner with a 64-channel head coil (Siemens, Erlangen, Germany). For patients with post-COVID syndrome and healthy controls, we acquired a high-resolution structural T1-weighted scan (3D-MPRAGE, TR = 1900 ms, TE = 2.22 ms, TI = 2100 ms, voxel size 1 × 1 × 1 mm³), as well as a diffusion scan (multiband EPI, 98 diffusion directions, b = 3000 s/mm^2^, 1.5 mm slice thickness, voxel size 1.5 × 1.5 × 1.5 mm³). For patients with MS, we acquired a 3D-MPRAGE (TR = 2500 ms, TE = 2.22 ms, TI = 2100 ms, voxel size 0.8 × 0.8 × 0.8 mm³), a 3D-FLAIR (TR = 6000 ms, TE = 387 ms, TI = 2100 ms, voxel size 0.8 × 0.8 × 0.8 mm³), and a diffusion scan (identical). Following N4-bias correction, and T1-coregistration, two expert MRI technicians (>10 years of experience) manually segmented T2-hyperintense lesions using ITK-SNAP (www.itksnap.org) from FLAIR images. Lesion masks were used to in-fill MPRAGE scans using FSL lesion_filling.

### Imaging data analysis

Diffusion images were preprocessed using the diffusion toolbox in FSL (https://fsl.fmrib.ox.ac.uk/fsl/fslwiki/). Preprocessing included a visual quality check for artefacts, brain extraction, and correction for participant motion and distortions induced by eddy currents. We subsequently fitted the diffusion tensors to the data to obtain participant-specific maps for fractional anisotropy (FA), axial diffusivity (AD), radial diffusivity (RD), and mean diffusivity (MD). These maps were statistically compared between study groups on a voxel-by-voxel basis using tract-based spatial statistics (TBSS). Here, individual diffusion maps were nonlinearly registered to MNI152 standard space. After projecting the FA, AD, RD, and MD maps onto the mean skeleton, we performed group comparisons using FSL Randomise with 5000 permutations and corrected for multiple testing using threshold-free cluster enhancement.

Volume estimates of subcortical structures were obtained using model-based segmentation in FSL FIRST.^26^ The quality of automated segmentations was stringently visually controlled. Volumes were individually adjusted for a participant's whole brain volume (wBV) using the formula: *volume*_*adjusted*_ *= volume*_*observed*_ *− β [slope from wBV vs. regional volume regression] x (wBV*_*observed*_ *− wBV*_*sample mean*_*)*. We subsequently used these segmentations to create deformable surface meshes of the structures of interest using the vertex analysis implemented in FIRST. While volume scores provide a single, global metric for a brain structure, surface deformation mapping helps to plot pathological changes topographically. Hereby, shape analyses provide complementary information about the location of the shape changes that are associated with lower volumes. Surface deformations were compared between groups using FSL Randomise with TFCE and plotted using SurfIce (https://www.nitrc.org/projects/surfice/). Lastly, we used the segmentations of the individual structures to extract diffusion parameters from the preprocessed diffusion maps for each participant.

### Assessment of fatigue, sleep, neuropsychiatric symptoms, and cognitive function

Fatigue assessment included the physical and cognitive subscales of the FSMC, the Bell score for fatigue-related functional impairment, as well as the symptom questionnaire of the Canadian criteria for chronic fatigue syndrome/myalgic encephalomyelitis. Moreover, we evaluated sleep problems (Pittsburgh Sleep Quality Index, PSQI), daytime sleepiness (Epworth Sleepiness Scale, ESS), depressive symptoms (Beck Depression Inventory II, BDI-II), and anxiety levels (Beck Anxiety Inventory, BAI). Missing data points, if applicable, were handled according to the recommendations provided in the test manuals. Cognitive covariates included short-term memory performance (Rey Auditory Verbal Learning Test, RAVLT, first recall) and a dual-task paradigm (Testbatterie zur Aufmerksamkeitsprüfung, TAP, divided attention task). Test scores were compared to those of the healthy controls enrolled in this study to control for potential effects of general pandemic restrictions on well-being and cognitive function.

### Choice of primary measure

The Fatigue Scale for Motor and Cognitive Function (FSMC) was used as primary scale for the assessment of subjective fatigue severity. The German version of the FSMC is a widely used, freely available self-report questionnaire that was developed to assess physical and cognitive fatigue in patients with MS.^27^ The FSMC asks patients to rate their agreement to 20 items on a 5-point Likert Scale. Specifically, the FSMC allows to distinguish between physical (e.g., effects of fatigue on strength, speed, or resting periods; 10 items) and cognitive symptoms (e.g., effect on concentration, memory, or attention, 10 items). Cut-offs exist for the total score classifying mild (≥43), moderate (≥53), and severe fatigue (≥63), as well as for the individual subscales. The FSMC has an excellent test-retest reliability (r = 0.86), very good internal consistency (α > 0.91), and excellent convergent validity with the Fatigue Severity Scale (FSS, r = 0.80) and the Modified Fatigue Impact Scale (MFIS, r = 0.83).

### Statistical analysis

Hypothesis-based analyses included volumetric and diffusion imaging analyses of thalamus, basal ganglia, and striatum. Our experimental hypothesis assumed that post-COVID fatigue was associated with structural imaging alterations of one or more subcortical structures. After visual inspection of boxplots to identify potential outliers, we used paired t-tests to assess group differences between patients with post-COVID fatigue and healthy controls. P-values from hypothesis-driven analyses were adjusted for multiple comparisons using the Benjamini-Hochberg correction for each structure and hemisphere as implemented in the R package stats (version 3.6.2). Exploratory tests were only performed for structures with relevant group differences, i.e., thalamus, putamen and pallidum (volume, FA, MD, RD, AD). Correlations were tested for seven fatigue measures (FSMC-cog, FSMC-phys, FSMC-total, Bell, Canadian criteria, PSQI and ESS) yielding 210 correlations, two cognitive tests (RAVLT first recall and TAP divided attention) yielding 60 correlations and two psychosocial scales (BDI, BAI), also yielding 60 correlations.

Lastly, we used a multiple linear regression analysis to identify which factors of the acute COVID-19 infection and postacute phase are associated with post-COVID fatigue. Following the Shapiro–Wilk normality and log-transformation, if applicable, our model included clinical and neuropsychiatric variables (as presented in Fig. 4) and was controlled for multicollinearity. P-values represent 2-sided significance tests with an alpha level of α = 0.05. Statistical analyses were performed using R 4.1.2.

**Fig. 1:**
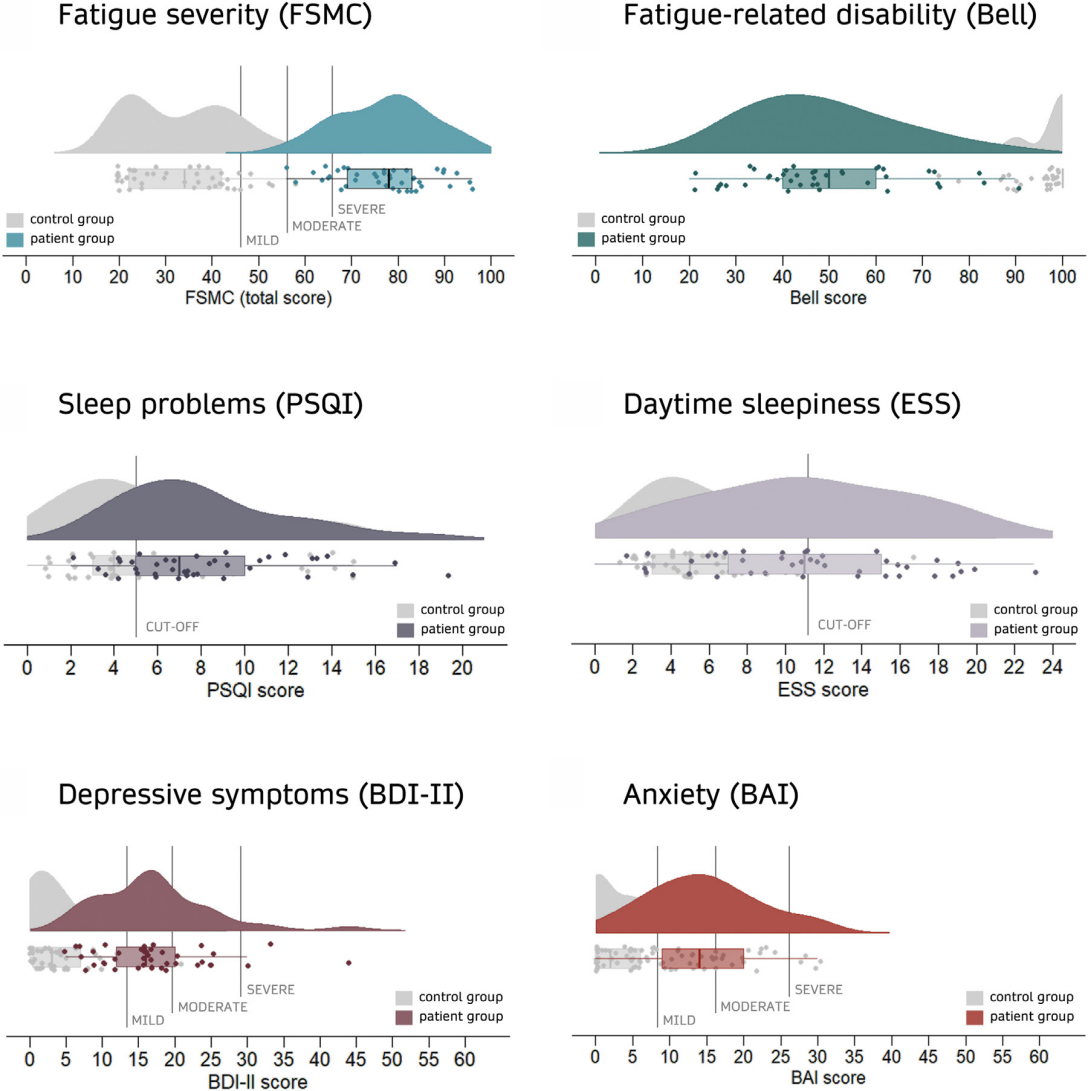
**High prevalence of concurrent sleep problems, depressive and anxiety symptoms in post-COVID patients with fatigue.** (BAI: Beck Anxiety Inventory, BDI-II: Beck Depression Inventory II, ESS: Epworth Sleepiness Scale, FSMC: Fatigue Scale for Motor and Cognitive Function, PSQI: Pittsburgh Sleep Quality Index).

**Fig. 2:**
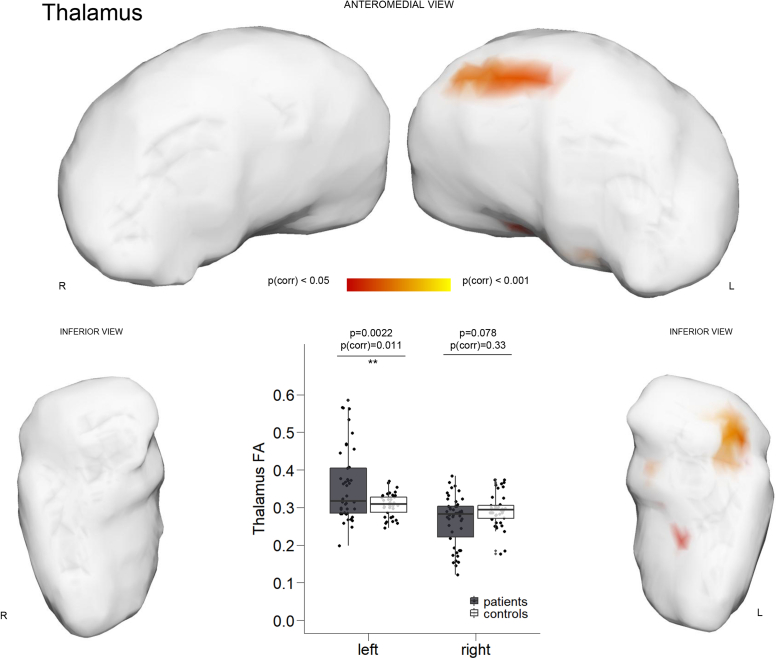
**Structural thalamic changes in patients with post-COVID fatigue.** Patients with moderate or severe fatigue after COVID-19 showed significantly higher thalamic fractional anisotropy (FA) and surface deformations (boxplots with whiskers extending to 1.5∗IQR).

**Fig. 3:**
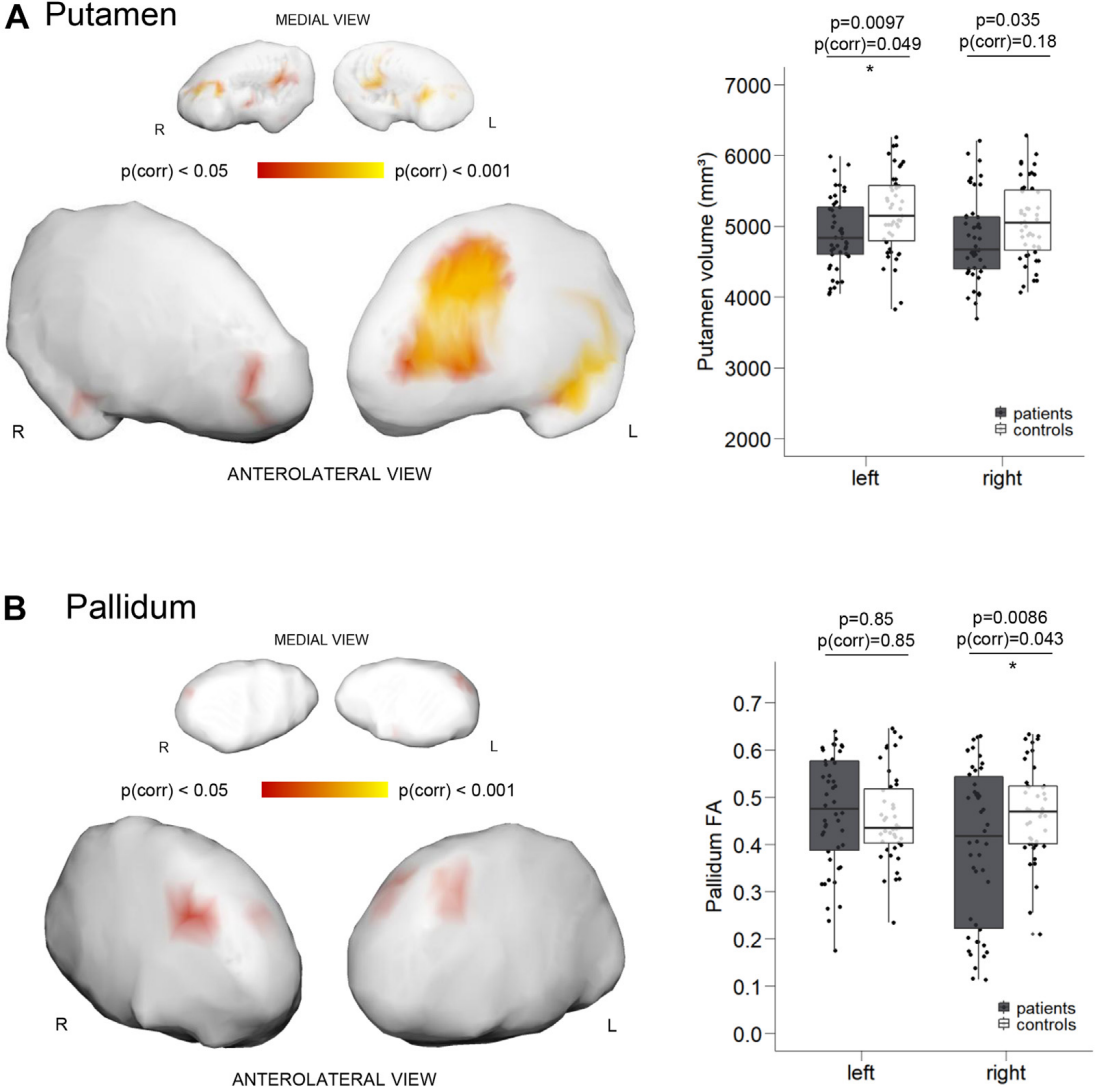
**Structural changes in the putamen (A) and pallidum (B) in patients with post-COVID fatigue.** Marked surface deformations, volume loss, and aberrant diffusion parameters show involvement of the basal ganglia in long-term fatigue after COVID-19 (boxplots with whiskers extending to 1.5∗IQR).

**Fig. 4:**
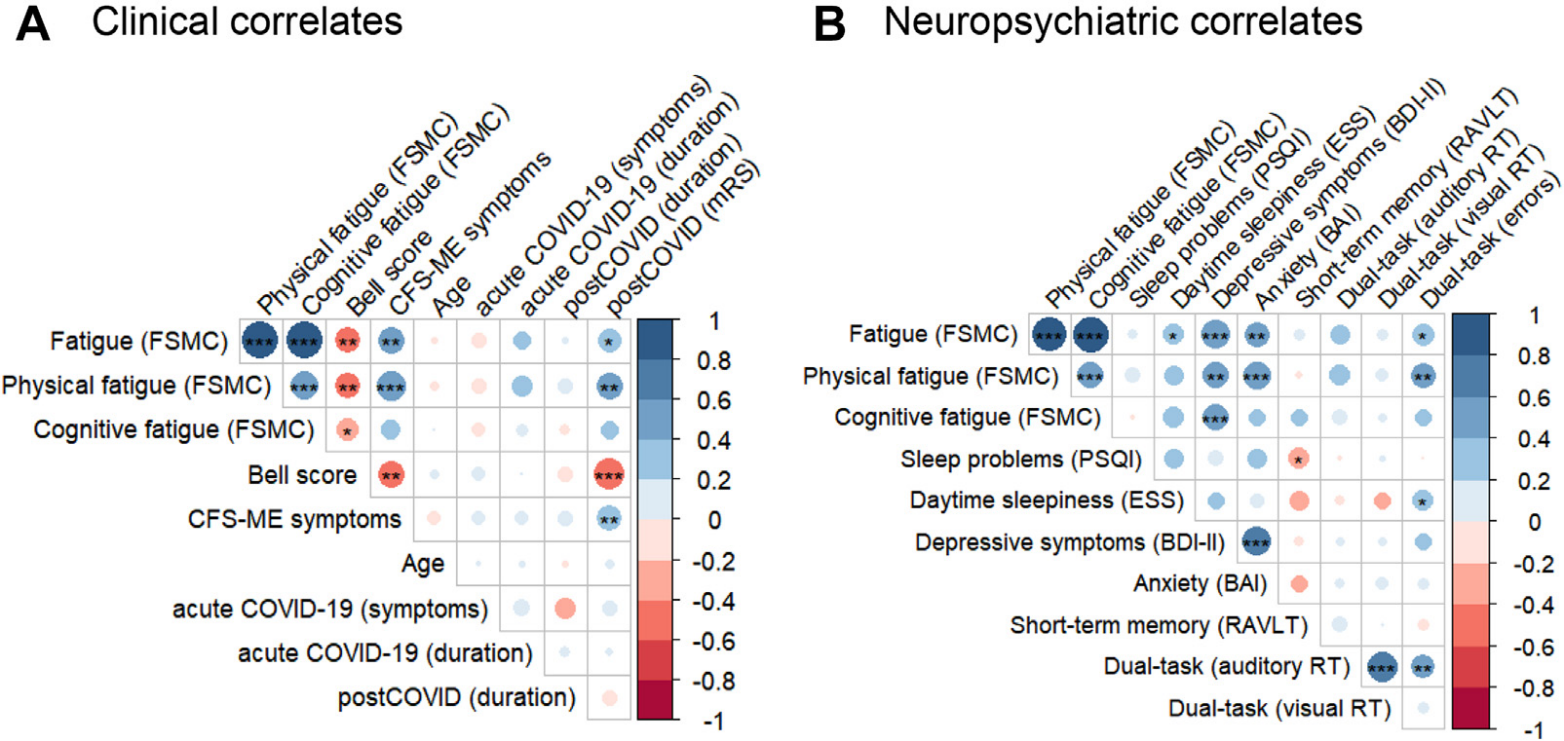
**Correlation plots of clinical parameters, neuropsychiatric scales, and cognitive tests in patients with post-COVID fatigue. A.** Long-term fatigue was unrelated to the severity of the acute COVID-19 disease. **B.** Instead, post-COVID fatigue was associated with higher depressiveness, anxiety, and an increased susceptibility to errors on a test of higher attention. Post-COVID disease duration is defined as the time between the onset of COVID-19 and study assessment. (BAI: Beck Anxiety Inventory, BDI-II: Beck Depression Inventory II, CFS-ME: chronic fatigue syndrome/myalgic encephalomyelitis – consensus criteria symptom count, ESS: Epworth Sleepiness Scale, FSMC: Fatigue Scale for Motor and Cognitive Function, mRS: modified Rankin Scale, PSQI: Pittsburgh Sleep Quality Index, RAVLT: Rey Auditory Verbal Learning Test, RT: response time ∗p < 0.05, ∗∗p < 0.01, ∗∗∗p < 0.001).

### Role of the funding source

The funding source had no role in study design, data collection, data analysis, data interpretation, or writing of the report. All authors had access to the dataset used in this study. J.H. and C.F. had final responsibility for the decision to submit this manuscript for publication.

## Results

### Clinical characteristics

Detailed clinical information about the acute COVID-19 disease stage is presented in Table 1. Patients were predominantly female (39/47). The previous medical history in this patient sample included asthma (6/47, 13%), allergies/atopic dermatitis (6/47, 13%), hypertension (5/47, 11%), hypothyroidism (4/47, 9%), coagulation disorder (2/47, 4%), and breast cancer (1/47). Two patients reported infrequent migraine. In addition, three patients reported having had episodes of depression, anxiety and eating disorder throughout their lifetime. Since the onset of COVID-19, four patients had new-onset hypertension, two were diagnosed with asthma, and one patient developed heart problems, including arrhythmias and angina pectoris.

At the time of the study visit, spontaneously reported complaints were feeling exhausted (39/47, 83%), difficulty concentrating (39/47, 83%), forgetfulness (29/47, 62%), feeling tired (26/47, 55%), word finding difficulties (21/47, 45%), headache (19/47, 40%), and feeling stressed/less resilient (17/47, 36%). In contrast, respiratory or systemic inflammatory symptoms were less common and included persistent dyspnoea (12/47, 26%), muscle or joint pain (9/47, 19%), chest pain (6/47, 13%), and flu-like symptoms (5/47, 11%) in some patients.

### Neuropsychiatric and cognitive characteristics

Both physical and cognitive fatigue were similarly affected in patients with post-COVID fatigue (Table 1). Additionally, we observed increased levels of depressive symptoms (BDI-II: 17.0 (7.8) vs. 4.8 (5.4), Hedges’ g [95% CI] = 1.11 [0.73, 1.51], p < 0.0001), anxiety (BAI: 14.6 (7.5) vs. 3.9 (4.8), g = 1.06 [0.68, 1.45], p < 0.0001), daytime sleepiness (ESS: 11.2 (5.6) vs. 5.5 (3.2), g = 0.8 [0.46, 1.16], p < 0.0001), and sleep problems (PSQI: 8.2 (3.9) vs. 5.8 (4.1), g = 0.38 [0.07, 0.7], p = 0.0074) compared to healthy controls (Fig. 1). The most affected PSQI subscales were daytime dysfunction (1.85 (0.79)), subjective sleep quality (1.54 (0.78)), and sleep disturbances (1.46 (0.60)). On the test of higher attention, patients with post-COVID fatigue showed slowed response times (visual: 777 (83)ms vs. 710 (60)ms, g = 0.62 [0.28, 0.97], p = 0.00037; auditory: 628 (133)ms vs. 533 (83)ms, g = 0.55 [0.24, 0.88], p = 0.00052), while maintaining adequate accuracy (errors: 2.4 (1.8) vs. 1.9 (1.9), g = 0.15 [−0.16, 0.45], p = 0.34).

### Thalamic structural changes

The analysis of subcortical structures revealed several aberrant structural imaging markers (Supplementary Materials Table S1). Shape-based analyses revealed a significant inward deformation of the left thalamic surface in patients as shown in Fig. 2 (p = 0.018). These surface inversions affected a cluster on the surface area of the medial nuclear group (approximately covering the mediodorsal and midline thalamic nuclei), as well as an area on the inferior surface (approximately covering the ventral anterior and ventral lateral nuclei). When exploring associations with cognitive deficits, lower thalamic volumes were associated with a lower short-term memory performance on a word list-learning paradigm (r = 0.409, p = 0.0071, Supplementary Materials Fig. S1).

Next, we quantified diffusion parameters to assess microstructural tissue alterations. We observed altered fractional anisotropy of the left thalamus (Hedges’ g [95% CI] = 0.55 [0.2, 0.91], p = 0.0022, p_BH-corrected_ = 0.011; Fig. 2A.). In exploratory analyses, aberrant thalamic diffusion parameters were associated with physical fatigue (FSMC motor subscale: r = −0.413, p = 0.0081) and fatigue-related day-to-day disability (Bell score: r = −0.404, p = 0.012; Supplementary Materials Fig. S1).

### Basal ganglia structural changes

Volumetric analyses showed reduced volumes in the left putamen (g = −0.4, [−0.7, −0.1], p = 0.0097, p_BH-corrected_ = 0.049) in patients with post-COVID fatigue. These were accompanied by marked surface deformations, particularly on the lateral surface of the left putamen (p = 0.012; right: p = 0.014; Fig. 3A). In exploratory analyses, volume loss in the right putamen was related to fewer recalled words on a short-term memory paradigm (r = 0.392, p = 0.010; Supplementary Materials S1). Similarly, a shape analysis revealed significant surface deformations on the anterior portion of the left (p = 0.040) and right pallidum (p = 0.044; Fig. 3A). Structural alterations additionally encompassed reduced right pallidal FA, (g = −0.42 [−0.74, −0.11], p = 0.0086, p_BH-corrected_ = 0.043). When exploring associations with questionnaire data, aberrant pallidal diffusion parameters were associated with fatigue (FSMC: r = −0.336, p = 0.037) and daytime sleepiness (ESS: r = 0.313, p = 0.049, Supplementary Materials Fig. S1).

Apart from the affected thalamic and basal ganglia structures, we observed no significant imaging changes of the accumbens nucleus or caudate nucleus in this sample of patients with moderate to severe fatigue.

### White matter integrity

Tract-based spatial statistics revealed no differences in fractional anisotropy, axial diffusivity, radial diffusivity, or mean diffusivity on the whole brain level in patients with post-COVID fatigue compared to healthy controls.

### Factors associated with post-COVID fatigue

In a multiple linear regression analysis, sleep quality (β = −0.48, p = 0.014) and depressiveness (β = 0.52, p = 0.049) were associated with post-COVID fatigue (R^2^ = 0.81, F(9,12) = 5.62, p = 0.0041). In contrast, fatigue severity was unrelated to variables of the acute COVID-19 disease, including sex (female: 77.2 (10.4), male: 76.4 (9.4), t(9.3) = 0.19, p = 0.86), age at onset (r = −0.028, p = 0.86), the duration (r = 0.227, p = 0.17) and number of symptoms during acute COVID-19 (r = −0.120, p = 0.58) and the duration of post-COVID symptoms (r = 0.030, p = 0.85; Fig. 4A).

Long-term physical fatigue, however, was associated with higher neurological disability as assessed using the modified Rankin Scale (r = 0.461, p = 0.0038). Both cognitive (r = −0.331, p = 0.037) and physical fatigue scores (r = −0.404, p = 0.0098) were related to greater functional impairment in daily activities (Bell Score). Additionally, fatigue severity correlated with higher levels of depressiveness (r = 0.577, p < 0.0001), anxiety (r = 0.430, p = 0.0050), and daytime sleepiness (r = 0.337, p = 0.031, Fig. 4B) and was associated with a greater susceptibility to errors on a dual-task attention paradigm (TAP: r = 0.380, p = 0.014).

### Comparative analysis in patients with multiple sclerosis and fatigue

Lastly, we analysed how structural brain changes observed in patients with post-COVID fatigue compared to those seen in patients with MS-related fatigue. Patients with MS had a mean lesion count of 65.8 (58.2) with a mean total lesion volume of 9561 (12,291) mm³. Compared to healthy controls, we observed significantly lower volumes of the bilateral thalamus, putamen, and accumbens, as well as unilateral reductions of the left caudate and pallidum (all p < 0.01, Table S2). Microstructural changes were pronounced and largely affected the thalamus and basal ganglia areas (Supplementary Materials Table S2). Fatigue scores were strongly associated with disability (EDSS, r = 0.54, p = 0.0001). Structural changes of the putamen (*volume*, r = −0.32, p = 0.030) and thalamus (*volume*, r = −0.464, p = 0.0013; *FA*, r = 0.419, p = 0.0037) were associated with lesion load, but not directly with fatigue.

## Discussion

Our study shows that post-COVID fatigue is associated with distinct structural brain alterations in subcortical hubs that are detectable using MRI. Specifically, we identified reduced volumes and aberrant diffusion markers of the thalamus and basal ganglia that correlated with fatigue severity and impairment in daily activities, daytime sleepiness, and short-term memory problems. Importantly, this pattern of pathological changes emerged even though this cohort is relatively young, most patients were not hospitalised during their acute infection, and patients were in overall good health before COVID-19. Our novel finding - that post-COVID fatigue is associated with structural brain damage - highlights the importance of consequent therapeutic management of this debilitating postinfectious syndrome.

Fatigue is one of the most common and most detrimental sequelae of COVID-19.^11^^,^^28^ Around 20–30% of patients with COVID-19 experience fatigue at 12 weeks after their acute infection – independent of a previous hospitalisation.^12^^,^^13^ In line with this, we found that fatigue severity was independent of the severity of the acute disease course, such as the duration of COVID-19 and the number of acute symptoms. In contrast, post-COVID fatigue was strongly associated with the manifestation of depressive symptoms and sleep quality several months after the acute infection. Although fatigue is a subjective symptom based on self-report,^6^ it has substantial and relevant consequences for a patient's everyday life. The continuous experience of overwhelming exhaustion and low energy levels impacts quality of life,^11^ and around 30–50% of patients are unable to return to their previous workplace.^12^ Results on associated risk factors are heterogeneous, but accumulating evidence suggests that early neurological involvement may be an important precondition of long-term fatigue.^13^^,^^29^

The structural imaging changes in the thalamus and basal ganglia observed in this study are the first description of fatigue-related brain correlates in patients with post-COVID syndrome. This distinct pattern of subcortical involvement is consistent with evidence from other neurological disorders associated with fatigue.^7^ In patients with multiple sclerosis and fatigue, magnetic resonance imaging and positron emission tomography studies have consistently pointed to alterations of cortico-subcortical pathways, also involving the thalamus, basal ganglia, and prefrontal cortex.^6^ Specifically, imaging correlates in fatigued patients with MS include atrophy, reduced perfusion, reduced glucose metabolism, increased activation during motor tasks, and disrupted functional connectivity.^6^^,^^20^^,^^30^

In this study of patients with post-COVID fatigue, we observed significant volume reductions of the left thalamus and bilateral putamen that were accompanied by marked surface inversions seen in a shape analysis. Moreover, aberrant diffusion markers in the thalamus and right pallidum correlated with fatigue severity, daytime sleepiness, and the extent of fatigue-related disability in everyday life. Furthermore, prominent fatigue after administration of interferon (IFN)-α was found to be associated with higher basal ganglia metabolism.^31^ These findings suggest that a distinct pattern of pathological brain changes gives rise to fatigue symptoms across diseases, similar to previous descriptions of shared correlates of memory dysfunction in neuroimmunological disorders.^24^ The molecular pathophysiology underlying fatigue is, however, still unclear. Although studies reporting cytokine and endocrine abnormalities suggest an immune-mediated mechanism,^31^ there is currently no effective cell-based or serum marker available for clinical practice.^6^

The basal ganglia support a wide range of functions beyond motor control, including memory, motivation, and reward-guided behaviour. Recently, it has been suggested that effort-reward imbalance, i.e., a biased perception towards high performance costs and low benefits, is a key factor in fatigue pathogenesis.^7^^,^^32^ Indeed, this perception bias was linked to alterations of basal ganglia functional connectivity and dysfunction of the cortico-striatal networks.^20^^,^^32^ In particular, a disruption of dopaminergic basal ganglia-circuits may be a major neural substrate of fatigue and has been referred to as the dopamine imbalance hypothesis of fatigue.^32^ Given our current findings on basal ganglia damage and the accumulating evidence of a neuroimmunological aetiology,^33^ a disruption of cortico-striatal circuits together with dopaminergic imbalance in the basal ganglia might contribute to post-COVID fatigue.

Our clinical control group of patients with MS and fatigue showed more widespread subcortical changes than those seen in patients with post-COVID syndrome. This overlap in affected brain regions points to a potential shared neuronal substrate of fatigue symptoms between diseases. Subcortical changes in MS-fatigue were, however, more strongly associated with lesion load than fatigue score themselves. Given that MS is a chronic-inflammatory disease of the central nervous system with a wide set of neurological and cognitive symptoms, the interplay between demyelinating and neurodegenerative processes is likely to be more complex than in a postviral fatigue.

In addition to basal ganglia involvement, altered thalamic function has gained more attention as a correlate of fatigue throughout the last years.^34^ In a study using [18F] FDG-PET imaging in patients with early relapsing-remitting MS, lower thalamic glucose metabolism at rest was associated with higher fatigue.^35^ Moreover, fatigued patients with MS showed lower cerebral blood flow in the thalamus, putamen, and caudate compared to healthy controls.^36^ In the latter perfusion MRI study, higher fatigue was related to lower cerebral blood flow and cerebral blood volume. Indeed, given its role as a hub-like gateway relaying sensory and motor information between cortical and subcortical areas, it is conceivable that disease-related changes to thalamic structure or function contribute to fatigue-related and cognitive symptoms.

In our study cohort, the most common subjective complaints of patients with post-COVID fatigue were exhaustion (83%), difficulties to concentrate (83%), and forgetfulness (62%). These complaints were reported by patients during study interviews and mirror the fatigue correlates obtained using standardised neuroimaging assessments. For instance, aberrant diffusion markers in the thalamus and pallidum were related to fatigue scores on validated clinical scales. Moreover, reduced thalamic and putamen volumes were associated with worse verbal short-term memory on a word list-learning paradigm. On a test of higher attention, patients with higher fatigue scores committed more errors when asked to perform two tasks simultaneously. This difficulty in allocating attentional resources to complex tasks quantifies the subjective experience of struggling with concentration in everyday life.

Some of the main strengths of this study are its prospective design and its stringent inclusion criteria. All included patients had documentation of a positive PCR test for COVID-19, fulfilled the international WHO consensus criteria for post-COVID syndrome,^3^ and underwent standardised neuropsychiatric and cognitive assessments. The high proportion of female patients in our study sample (39/47 patients) reflects the higher prevalence of fatigue in women that has also been reported in post-COVID syndrome.^13^^,^^37^ Although the mechanisms underlying these sex differences are not yet fully understood, a higher susceptibility to immune-mediated diseases may be a key factor.^38^

At a median of 7.5 (IQR 6.5–9.2) months after the acute infection, the subjective fatigue presentation in this sample spanned cognitive and motor fatigue equally. Future studies may determine how the subjective experience of central fatigue relates to peripheral measures of performance fatiguability. Additionally, we observed that post-COVID fatigue is accompanied by increased levels of daytime dysfunction, sleep problems, anxiety, and depressiveness that further add to the disease burden. It could be argued that these symptoms may arise from general pandemic-related stressors such as social isolation or financial insecurity rather than the virus infection itself. However, our control group was well matched regarding age, sex and education and assessed during the same period in the same city as the patient group and therefore exposed to the same socio-economic and societal stressors. Consequently, the here observed group differences of fatigue-related changes in mood, energy levels, and sleep are very likely attributable to post-COVID syndrome.

This study has several limitations: (1) This study underlies the general limitations of cross-sectional designs. Longitudinal studies are needed to determine the duration and course of the symptoms, and ultimately inform the prognosis of post-COVID syndrome. (2) Results from our exploratory analyses are to be interpreted with caution. Due to the novelty of the disease, the purpose of these analyses was to generate new hypotheses for future studies. As such, the observed correlations we found will need to be reproduced in other samples.”

In conclusion, our analyses show that a distinct pattern of thalamic and basal ganglia changes is associated with post-COVID fatigue. Imaging alterations include volume reductions, surface deformations, and aberrant diffusion markers that correlate with the severity and everyday impact of fatigue symptoms. Moreover, we show that post-COVID fatigue needs to be managed in a wider clinical array that also considers sleep quality, mood alterations, and cognitive impairment. Future research will determine whether these fatigue symptoms are transient or persistent. The identification of distinct subcortical brain correlates provides a foundation for further research on the pathomechanisms of post-COVID fatigue.

## Contributors

J.H. and C.Fi. designed the study. J.H., K.S., C.C., F.B., R.R., J.B.-S., and C.Fr. acquired the data. J.H., K.S., S.R., T.J.H. and C.C. analysed the data. J.H., T.J.H., and C.Fi. drafted the manuscript. K.S., S.R., C.C., F.B., R.R., C.H., J.B., A.S.C., C.S., J.B.-S., F.P., C.Fr., and K.R. revised the manuscript for intellectual content. All authors had access to and verified the dataset used in this study.

## Data sharing statement

Deidentified imaging data are available on request from the corresponding author.

## Declaration of interests

Dr. Chien reports personal fees from Bayer, grants from Novartis, outside the submitted work. Dr. Bellmann-Strobl reports personal fees from Bayer Healthcare, personal fees from sanofi-aventis/Genzyme, personal fees from Roche, outside the submitted work. Dr. Paul reports personal fees and non-financial support from SanofiGenzyme, personal fees, non-financial support and other from BiogenIdec, personal fees and non-financial support from MedImmune, personal fees and non-financial support from Shire, personal fees and non-financial support from Alexion, grants, personal fees and non-financial support from Bayer, grants and personal fees from Novartis, grants and personal fees from Teva, grants and personal fees from Merck Serono, personal fees from Actelion, personal fees from Chugai, personal fees from Roche, personal fees from Celgene, grants from Sanofi-Aventis/Genzyme, grants from Alexion, grants from German Research Council (DFG Exc 257), grants from Werth Stiftung of the City of Cologne, grants from German Ministry of Education and Research (BMBF Competence Network Multiple Sclerosis), grants from Arthur Arnstein Stiftung Berlin, grants from EU FP7 Framework Program (combims.eu), grants from Guthy Jackson Charitable Foundation, grants from National Multiple Sclerosis Society of the USA, outside the submitted work. All other authors declare no competing interests.

## Appendix A Supplementary data

Tables S1, S2 and Figure S1

STROBE Statement
