# Supplementary material for: Structural brain changes in patients with post-COVID fatigue: a prospective observational study
Source: eClinicalMedicine. 2023 Feb 27;58:101874. doi: 10.1016/j.eclinm.2023.101874 (PMC9969172; doi:10.1016/j.eclinm.2023.101874)
Supplement: Tables S1, S2 and Figure S1 [file mmc1.pdf]

## Supplementary material

**Table S1. Structural integrity of subcortical structures in patients with post-COVID fatigue compared to matched healthy controls.** (AD: axial diffusivity, FA: fractional anisotropy, MD: mean diffusivity, RD: radial diffusivity, BH: Benjamini-Hochberg, * p(BH-adjusted) < 0.05)

| Structure | Hemisphere | Marker | Hedges‘ g | Paired t(df) | p | p (BH-adjusted) |
|---|---|---|---|---|---|---|
| Accumbens | left | Volume | -0.25, [-0.55, 0.04] | t(44) = -1.74 | 0.089 | 0.26 |
| | | FA | 0.06, [-0.23, 0.35] | t(44) = 0.43 | 0.67 | 0.84 |
| | | MD | -0.24, [-0.54, 0.05] | t(44) = -1.67 | 0.1 | 0.26 |
| | | AD | 0.03, [-0.26, 0.32] | t(43) = 0.20 | 0.84 | 0.84 |
| | | RD | -0.17, [-0.46, 0.12] | t(44) = -1.17 | 0.25 | 0.42 |
| | right | Volume | -0.24, [-0.53, 0.06] | t(44) = -1.61 | 0.11 | 0.47 |
| | | FA | 0.03, [-0.26, 0.33] | t(44) = 0.24 | 0.81 | 0.81 |
| | | MD | -0.16, [-0.45, 0.14] | t(44) = -1.06 | 0.29 | 0.49 |
| | | AD | 0.1, [-0.2, 0.4] | t(41) = 0.63 | 0.53 | 0.66 |
| | | RD | -0.2, [-0.49, 0.1] | t(44) = -1.34 | 0.19 | 0.47 |
| Caudate | left | Volume | -0.2, [-0.5, 0.09] | t(43) = -1.36 | 0.18 | 0.54 |
| | | FA | 0.12, [-0.17, 0.41] | t(44) = 0.80 | 0.43 | 0.54 |
| | | MD | -0.07, [-0.36, 0.22] | t(44) = -0.50 | 0.62 | 0.62 |
| | | AD | -0.14, [-0.43, 0.15] | t(44) = -0.95 | 0.35 | 0.54 |
| | | RD | -0.16, [-0.45, 0.13] | t(44) = -1.08 | 0.28 | 0.54 |
| | right | Volume | -0.02, [-0.31, 0.27] | t(45) = -0.15 | 0.88 | 0.88 |
| | | FA | 0.05, [-0.25, 0.34] | t(43) = 0.31 | 0.76 | 0.88 |
| | | MD | -0.1, [-0.41, 0.21] | t(38) = -0.63 | 0.53 | 0.88 |
| | | AD | -0.07, [-0.37, 0.23] | t(42) = -0.47 | 0.64 | 0.88 |
| | | RD | -0.28, [-0.6, 0.03] | t(39) = -1.81 | 0.079 | 0.39 |
| Pallidum | left | Volume | 0.04, [-0.26, 0.34] | t(41) = 0.24 | 0.81 | 0.85 |
| | | FA | 0.03, [-0.27, 0.33] | t(42) = 0.19 | 0.85 | 0.85 |
| | | MD | 0.18, [-0.11, 0.48] | t(44) = 1.25 | 0.22 | 0.44 |
| | | AD | -0.17, [-0.47, 0.13] | t(42) = -1.13 | 0.27 | 0.44 |
| | | RD | 0.19, [-0.1, 0.48] | t(44) = 1.29 | 0.2 | 0.44 |
| | right | Volume | 0.01, [-0.29, 0.31] | t(42) = 0.08 | 0.94 | 0.94 |
| | | FA | -0.42, [-0.74, -0.11] | t(40) = -2.76 | 0.0086 | 0.043 * |

| | | | | | | |
|---|---|---|---|---|---|---|
| | | MD | 0.07, [-0.22, 0.36] | t(44) = 0.48 | 0.63 | 0.94 |
| | | AD | -0.02, [-0.31, 0.28] | t(42) = -0.11 | 0.91 | 0.94 |
| | | RD | 0.2, [-0.09, 0.5] | t(44) = 1.39 | 0.17 | 0.43 |
| Putamen | left | Volume | -0.4, [-0.7, -0.1] | t(44) = -2.70 | 0.0097 | 0.049 * |
| | | FA | 0.07, [-0.22, 0.36] | t(44) = 0.47 | 0.64 | 0.89 |
| | | MD | -0.02, [-0.32, 0.28] | t(42) = -0.14 | 0.89 | 0.89 |
| | | AD | 0.03, [-0.3, 0.35] | t(35) = 0.16 | 0.87 | 0.89 |
| | | RD | 0.05, [-0.26, 0.36] | t(39) = 0.30 | 0.76 | 0.89 |
| | right | Volume | -0.32, [-0.62, -0.02] | t(44) = -2.17 | 0.035 | 0.18 |
| | | FA | -0.18, [-0.48, 0.11] | t(44) = -1.26 | 0.22 | 0.27 |
| | | MD | 0.2, [-0.1, 0.49] | t(44) = 1.34 | 0.19 | 0.27 |
| | | AD | 0.17, [-0.15, 0.5] | t(36) = 1.07 | 0.29 | 0.29 |
| | | RD | 0.22, [-0.07, 0.52] | t(44) = 1.50 | 0.14 | 0.27 |
| Thalamus | left | Volume | -0.32, [-0.62, -0.02] | t(44) = -2.16 | 0.036 | 0.09 |
| | | FA | 0.55, [0.2, 0.91] | t(34) = 3.32 | 0.0022 | 0.011 * |
| | | MD | 0.17, [-0.12, 0.47] | t(44) = 1.19 | 0.24 | 0.40 |
| | | AD | -0.06, [-0.35, 0.23] | t(44) = -0.39 | 0.70 | 0.80 |
| | | RD | 0.04, [-0.25, 0.33] | t(44) = 0.25 | 0.80 | 0.80 |
| | right | Volume | -0.22, [-0.52, 0.07] | t(45) = -1.54 | 0.13 | 0.33 |
| | | FA | -0.28, [-0.6, 0.03] | t(38) = -1.81 | 0.078 | 0.33 |
| | | MD | -0.06, [-0.35, 0.23] | t(44) = -0.43 | 0.67 | 0.83 |
| | | AD | 0, [-0.29, 0.29] | t(44) = 0.03 | 0.98 | 0.98 |
| | | RD | -0.1, [-0.41, 0.2] | t(40) = -0.67 | 0.50 | 0.83 |

**Table S2. Structural integrity of subcortical structures in MS patients with fatigue compared to matched healthy controls.** (AD: axial diffusivity, FA: fractional anisotropy, MD: mean diffusivity, RD: radial diffusivity, * p(BH-adjusted) < 0.05, ** p(BH-adjusted) < 0.01, *** p(BH-adjusted) < 0.001)

| Structure | Hemisphere | Marker | Hedges' g | Paired t(df) | p | p (BH-adjusted) |
|---|---|---|---|---|---|---|
| Accumbens | left | Volume | -0.69, [-1.02, -0.37] | t(44) = -4.70 | < 0.0001 | 0.00013 *** |
| | | FA | 0.06, [-0.23, 0.35] | t(45) = 0.40 | 0.69 | 0.69 |
| | | MD | -0.46, [-0.77, -0.15] | t(44) = -3.12 | 0.0032 | 0.0081 ** |
| | | AD | -0.27, [-0.57, 0.02] | t(45) = -1.89 | 0.066 | 0.082 |
| | | RD | -0.38, [-0.69, -0.08] | t(43) = -2.57 | 0.014 | 0.023 * |
| | right | Volume | -1.06, [-1.44, -0.69] | t(42) = -7.07 | < 0.0001 | < 0.0001 *** |
| | | FA | -0.02, [-0.31, 0.27] | t(44) = -0.16 | 0.87 | 0.87 |
| | | MD | -0.05, [-0.34, 0.24] | t(43) = -0.33 | 0.74 | 0.87 |
| | | AD | -0.09, [-0.39, 0.21] | t(42) = -0.60 | 0.55 | 0.87 |
| | | RD | -0.14, [-0.44, 0.15] | t(43) = -0.97 | 0.34 | 0.85 |
| Caudate | left | Volume | -0.74, [-1.09, -0.4] | t(40) = -4.85 | < 0.0001 | < 0.0001 *** |
| | | FA | -0.62, [-0.95, -0.3] | t(43) = -4.19 | 0.00014 | 0.00017 *** |
| | | MD | 0.91, [0.56, 1.26] | t(44) = 6.18 | < 0.0001 | < 0.0001 *** |
| | | AD | -0.6, [-0.92, -0.29] | t(44) = -4.08 | 0.00019 | 0.00019 *** |
| | | RD | 0.82, [0.49, 1.16] | t(44) = 5.58 | < 0.0001 | < 0.0001 *** |
| | right | Volume | -0.29, [-0.58, 0.01] | t(45) = -1.98 | 0.054 | 0.054 |
| | | FA | -0.49, [-0.81, -0.18] | t(42) = -3.25 | 0.0023 | 0.0028 ** |
| | | MD | 0.77, [0.41, 1.13] | t(37) = 4.82 | < 0.0001 | 0.00012 *** |
| | | AD | 0.58, [0.26, 0.9] | t(42) = 3.84 | 0.00041 | 0.001 ** |
| | | RD | 0.57, [0.22, 0.92] | t(35) = 3.48 | 0.0014 | 0.0023 ** |
| Pallidum | left | Volume | -0.46, [-0.78, -0.14] | t(40) = -2.99 | 0.0047 | 0.0059 ** |
| | | FA | -1.22, [-1.64, -0.83] | t(41) = -8.08 | < 0.0001 | < 0.0001 *** |
| | | MD | -0.95, [-1.31, -0.6] | t(43) = -6.39 | < 0.0001 | < 0.0001 *** |
| | | AD | -3.16, [-3.93, -2.44] | t(42) = -21.07 | < 0.0001 | < 0.0001 *** |
| | | RD | 0.11, [-0.19, 0.4] | t(43) = 0.72 | 0.47 | 0.47 |
| | right | Volume | -0.18, [-0.48, 0.12] | t(42) = -1.22 | 0.23 | 0.23 |
| | | FA | -1.07, [-1.46, -0.7] | t(41) = -7.05 | < 0.0001 | < 0.0001 *** |
| | | MD | -1.04, [-1.41, -0.68] | t(44) = -7.10 | < 0.0001 | < 0.0001 *** |
| | | AD | -1.92, [-2.44, -1.43] | t(43) = -12.93 | < 0.0001 | < 0.0001 *** |
| | | RD | -0.36, [-0.66, -0.06] | t(45) = -2.48 | 0.017 | 0.021 * |

| | | | | | | |
|---|---|---|---|---|---|---|
| Putamen | left | Volume | -1.03, [-1.41, -0.67] | t(43) = -6.98 | < 0.0001 | < 0.0001 *** |
| | | FA | -1.23, [-1.63, -0.85] | t(44) = -8.38 | < 0.0001 | < 0.0001 *** |
| | | MD | 0.4, [0.1, 0.71] | t(43) = 2.71 | 0.0096 | 0.0096 ** |
| | | AD | -2.42, [-3.08, -1.81] | t(37) = -15.21 | < 0.0001 | < 0.0001 *** |
| | | RD | 1.63, [1.18, 2.1] | t(42) = 10.87 | < 0.0001 | < 0.0001 *** |
| | left | Volume | -0.78, [-1.12, -0.45] | t(44) = -5.31 | < 0.0001 | < 0.0001 *** |
| | | FA | -1.23, [-1.63, -0.86] | t(45) = -8.51 | < 0.0001 | < 0.0001 *** |
| | | MD | 0.34, [0.05, 0.64] | t(45) = 2.38 | 0.022 | 0.022 * |
| | | AD | -0.54, [-0.88, -0.2] | t(37) = -3.37 | 0.0018 | 0.0022 ** |
| | | RD | 0.67, [0.36, 0.99] | t(45) = 4.63 | < 0.0001 | < 0.0001 *** |
| Thalamus | left | Volume | -1.04, [-1.41, -0.69] | t(46) = -7.27 | < 0.0001 | < 0.0001 *** |
| | | FA | 0.66, [0.3, 1.02] | t(35) = 4.04 | 0.00028 | 0.00047 *** |
| | | MD | -0.09, [-0.38, 0.2] | t(44) = -0.63 | 0.53 | 0.53 |
| | | AD | -1.33, [-1.75, -0.93] | t(43) = -8.99 | < 0.0001 | < 0.0001 *** |
| | | RD | 0.13, [-0.16, 0.42] | t(44) = 0.88 | 0.38 | 0.48 |
| | right | Volume | -1.06, [-1.44, -0.7] | t(44) = -7.25 | < 0.0001 | < 0.0001 *** |
| | | FA | 0.54, [0.21, 0.88] | t(38) = 3.42 | 0.0015 | 0.0019 ** |
| | | MD | -1.16, [-1.55, -0.79] | t(43) = -7.84 | < 0.0001 | < 0.0001 *** |
| | | AD | -0.28, [-0.58, 0.02] | t(42) = -1.85 | 0.072 | 0.072 |
| | | RD | -1.45, [-1.9, -1.03] | t(41) = -9.57 | < 0.0001 | < 0.0001 *** |

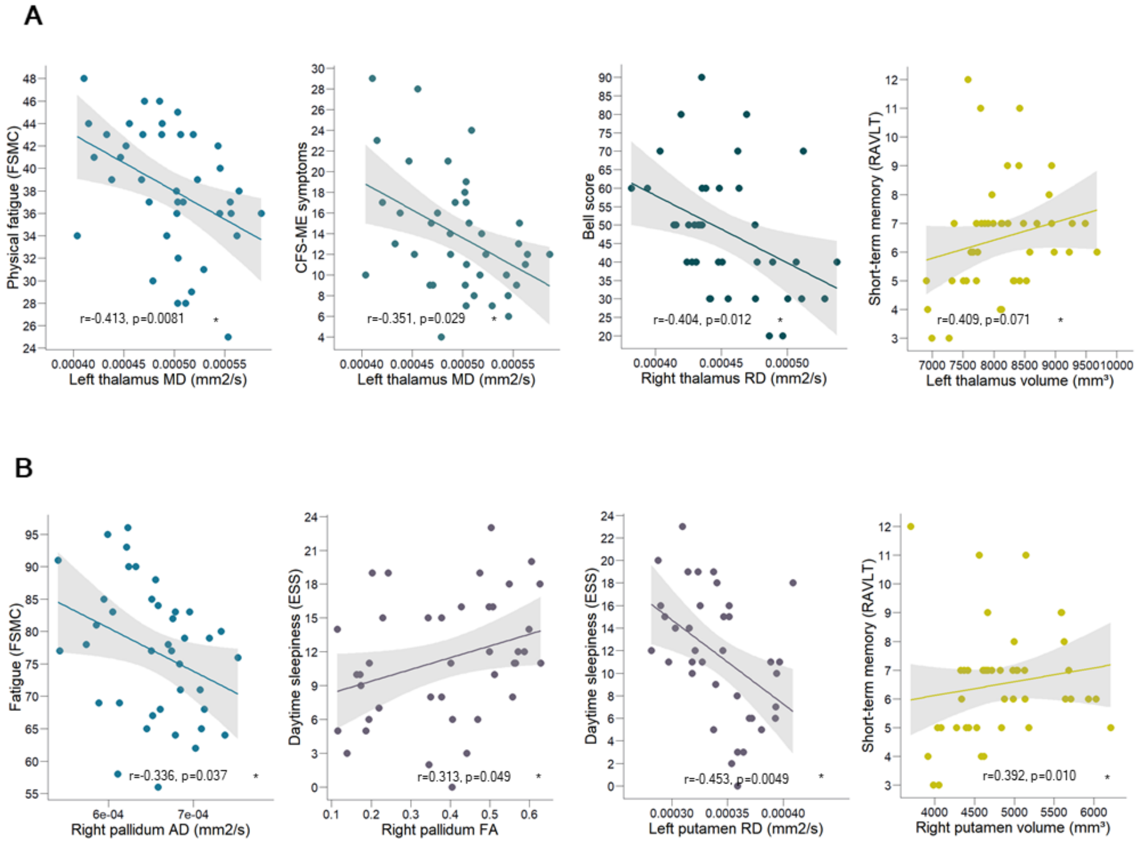


**Figure S1**. Correlation plots with 95% confidence interval. **A**. Lower thalamic volumes and altered diffusion characteristics were associated with fatigue severity and short-term memory performance. **B**. Diffusion parameters and volume of basal ganglia was related to worse memory performance, fatigue symptoms, and daytime sleepiness. **Abbreviations**: AD: axial diffusivity, CFS-ME: chronic fatigue syndrome/myalgic encephalomyelitis – consensus criteria symptom count, ESS: Epworth Sleepiness Scale, FA: fractional anisotropy, FSMC: Fatigue Scale for Motor and Cognitive Function, IQR: interquartile range, MD: mean diffusivity, RAVLT: Rey Auditory Verbal Learning Test, RD: radial diffusivity.
