# Supplementary material for: Structural brain changes in patients with post-COVID fatigue: a prospective observational study
Source: eClinicalMedicine. 2023 Feb 27;58:101874. doi: 10.1016/j.eclinm.2023.101874 (PMC9969172; doi:10.1016/j.eclinm.2023.101874)
Supplement: STROBE Statement [file mmc2.pdf]

STROBE Statement—checklist of items that should be included in reports of observational studies

	Item No.	Recommendation	Page No.	Relevant text from manuscript
**Title and abstract**	1	(a) Indicate the study's design with a commonly used term in the title or the abstract	page 2 (abstract)	"We prospectively recruited 50 patients from neurological post-COVID outpatient clinics (age 18-69 years, 39f/8m) and matched non-COVID healthy controls between April-December 2021."
		(b) Provide in the abstract an informative and balanced summary of what was done and what was found	page 2 (abstract)	"We prospectively recruited 50 patients from neurological post-COVID outpatient clinics (age 18-69 years, 39f/8m) and matched non-COVID healthy controls between April-December 2021. As a clinical control group, we included matched MS patients with fatigue. Assessments included diffusion and volumetric MR imaging, neuropsychiatric, and cognitive testing. At 7·5 months (median, IQR 6·5-9·2) after the acute SARS-CoV-2 infection, moderate or severe fatigue was identified in 47/50 post-COVID patients who were included in the analyses."<br><br>"Our diffusion imaging analyses revealed aberrant fractional anisotropy of the thalamus. Diffusion markers correlated with fatigue severity, such as physical fatigue, fatigue-related impairment in everyday life (Bell score) and daytime sleepiness. Moreover, we observed shape deformations and decreased volumes of the left thalamus, putamen, and pallidum which overlapped with the more extensive subcortical changes in MS and were associated with impaired short-term memory. Post-COVID fatigue severity was not related to COVID-19 disease courses (6/47 hospitalised, 2/47 with ICU treatment). In contrast, post-acute sleep quality and depressiveness emerged as significant determinants and were accompanied by increased levels of anxiety and daytime sleepiness compared to controls."
**Introduction**				
Background/rationale	2	Explain the scientific background and rationale for the investigation being reported	page 2 (abstract)<br><br>page 4 (introduction)	"Post-COVID syndrome is a severe long-term complication of COVID-19. Although fatigue and cognitive complaints are the most prominent symptoms, it is unclear whether they have structural correlates in the brain."<br><br>"In fact, cognitive complaints and fatigue are among the major neurological symptoms of patients with post-COVID syndrome and severely impact their quality of life.4 However, the pathomechanisms and imaging correlates of this novel disease entity still remain elusive."<br>"Previous research in neuroimmunological disorders identified robust associations of structural brain alterations with fatigue severity and cognitive impairment. For example, basal ganglia changes 20,21 and frontoparietal white matter damage 22 have consistently been linked to fatigue in multiple sclerosis, while hippocampal damage is associated with memory deficits across

				diseases.23,24 In the case of post-COVID syndrome, however, it remains currently unclear whether such distinct lesion patterns exist and whether structural brain damage underlies the persisting neurological symptoms in PCS patients."
Objectives	3	State specific objectives, including any prespecified hypotheses	page 2 (abstract)<br><br>pages 4-5 (introduction)	"We therefore explore the clinical characteristics of post-COVID fatigue, describe associated structural imaging changes, and determine what predicts post-COVID fatigue severity."<br><br>"Here, we (I) report the characteristics of persistent fatigue in a cohort of post-COVID patients; (II) analyse the integrity of white matter and subcortical structures using MR diffusion and volumetric imaging; (III) determine to what extent fatigue-related structural brain changes overlap with those observed in other aetiologies (i.e. multiple sclerosis); and (IV) determine whether features of the acute COVID-19 course predict subsequent post-COVID fatigue and associated structural brain changes."
**Methods**				
Study design	4	Present key elements of study design early in the paper	page 6 (methods)	"We prospectively recruited 50 patients from the neurological post-COVID outpatient clinic at Charité – Universitätsmedizin Berlin between April and November 2021. Inclusion criteria were (1) a history of confirmed SARS-CoV-2 infection (i.e., positive RT-PCR test) with (2) postinfectious neurological symptoms for at least 3 months and (3) no history of relevant neurological disease prior to COVID-19."
Setting	5	Describe the setting, locations, and relevant dates, including periods of recruitment, exposure, follow-up, and data collection	page 6 (methods)	"We prospectively recruited 50 patients from the neurological post-COVID outpatient clinic at Charité – Universitätsmedizin Berlin between April and November 2021. Inclusion criteria were (1) a history of confirmed SARS-CoV-2 infection (i.e., positive RT-PCR test) with (2) postinfectious neurological symptoms for at least 3 months and (3) no history of relevant neurological disease prior to COVID-19."
Participants	6	(a) Cohort study—Give the eligibility criteria, and the sources and methods of selection of participants. Describe methods of follow-up Case-control study—Give the eligibility criteria, and the sources and methods of case ascertainment and control selection. Give the rationale for the choice of cases and controls Cross-sectional study—Give the eligibility criteria, and the sources and methods of selection of participants	page 6 (methods)	"Inclusion criteria were (1) a history of confirmed SARS-CoV-2 infection (i.e., positive RT-PCR test) with (2) postinfectious neurological symptoms for at least 3 months and (3) no history of relevant neurological disease prior to COVID-19."<br>"Along with the post-COVID patient group, we prospectively recruited a healthy control group without history of neurological or psychiatric diseases and without previous COVID-19 infection. Healthy control participants were individually matched regarding age, sex, and education (n=47, mean age 44·5 (14·1) years) and received an identical study protocol."<br>"Moreover, we retrospectively identified a clinical control group of patients with multiple sclerosis and fatigue. Patients with MS were recruited between October 2019 and December 2021 at the NeuroCure Clinical Research Center (NCRC), Charité – Universitätsmedizin Berlin. Inclusion criteria were (1) a diagnosis of relapsing-remitting multiple sclerosis (RRMS) according to the McDonald 2017 diagnostic criteria and (2) moderate to severe fatigue (Fatigue Severy Scale (FSS)

				≥ 1 SD from healthy normative sample).25 MS patients were individually matched to post-COVID patients and healthy controls regarding age and sex (n=47, mean age 43·1 (10·7) years)."
			page 19 (discussion)	"Given the overall impact of the pandemic restrictions on public health and well-being, it could be argued that changes in sleep and mood are expected around 1·5-2 years into a global pandemic. To account for this, we studied carefully matched, non-COVID and non-fatigue controls between April and November 2021 that had experienced the same pandemic restrictions as the post-COVID patient group, causing e.g., social isolation, job insecurity and unhealthy work-life balance patterns. Consequently, the here observed group differences of fatigue-related changes in mood, energy levels, and sleep are very likely attributable to the post-COVID syndrome."
		(b) Cohort study—For matched studies, give matching criteria and number of exposed and unexposed<br>Case-control study—For matched studies, give matching criteria and the number of controls per case	page 6 (methods)	"Along with the post-COVID patient group, we prospectively recruited a healthy control group without history of neurological or psychiatric diseases and without previous COVID-19 infection. Healthy control participants were individually matched regarding age, sex, and education (n=47, mean age 44·5 (14·1) years) and received an identical study protocol."
Variables	7	Clearly define all outcomes, exposures, predictors, potential confounders, and effect modifiers. Give diagnostic criteria, if applicable	page 4 (introduction)	"This newly recognised syndrome has been defined as continued or new onset symptoms that persist for a least three months after COVID-19 and compromise everyday functioning.3"
			page 7 (methods)	"After projecting the FA, AD, RD, and MD maps onto the mean skeleton, we performed group comparisons using FSL Randomise with 5000 permutations and corrected for multiple testing using threshold-free cluster enhancement."<br>"Volume estimates of subcortical structures were obtained using model-based segmentation in FSL FIRST.26"<br>"Fatigue assessment included the physical and cognitive subscales of the FSMC, the Bell score for fatigue-related functional impairment, as well as the symptom questionnaire of the Canadian criteria for chronic fatigue syndrome/myalgic encephalomyelitis. Moreover, we evaluated sleep problems using the Pittsburgh Sleep Quality Index (PSQI), daytime sleepiness (Epworth Sleepiness Scale, ESS), depressive symptoms (Beck Depression Inventory II, BDI-II), and anxiety levels (Beck Anxiety Inventory, BAI). Cognitive covariates included short-term memory performance (Rey Auditory Verbal Learning Test, RAVLT, first recall) and a dual-task paradigm (Testbatterie zur Aufmerksamkeitsprüfung, TAP, divided attention task)."
			page 8 (methods)	"Choice of primary measure:<br>The Fatigue Scale for Motor and Cognitive Function (FSMC) was used as

				primary scale for the assessment of fatigue severity. The German version of the FSMC is a widely used, freely available self-report questionnaire that was developed to assess physical and cognitive fatigue in patients with MS.21 The FSMC asks patients to rate their agreement to 20 items on a 5-point Likert Scale. Specifically, the FSMC allows to distinguish between physical (e.g., effects of fatigue on strength, speed, or resting periods; 10 items) and cognitive symptoms (e.g., effect on concentration, memory, or attention, 10 items). Cut-offs exist for the total score classifying mild (≥43), moderate (≥53), and severe fatigue (≥63), as well as for the individual subscales. The FSMC has an excellent test-retest
Data sources/ measurement	8*	For each variable of interest, give sources of data and details of methods of assessment (measurement). Describe comparability of assessment methods if there is more than one group	page 2 (abstract)	"We prospectively recruited 50 patients from neurological post-COVID outpatient clinics (age 18-69 years, 39f/8m) and matched non-COVID healthy controls between April-December 2021. As a clinical control group, we included matched MS patients with fatigue. Assessments included diffusion and volumetric MR imaging, neuropsychiatric, and cognitive testing."
			page 7 (methods)	"For post-COVID patients and healthy controls, we acquired a high-resolution structural T1-weighted scan (3D-MPRAGE, TR=1900ms, TE=2·22 ms, TI=2100 ms, voxel size 1x1x1mm³), as well as a diffusion scan (multiband EPI, 98 diffusion directions, b=3000s/mm², 1·5mm slice thickness, voxel size 1·5x1·5x1·5mm³). For the MS patients, we acquired a 3D-MPRAGE (TR=2500ms, TE=2·22ms, TI=2100ms, voxel size 0·8x0·8x0·8mm³), a 3D-FLAIR (TR=6000ms, TE= 387ms, TI= 2100ms, voxel size 0.8x0.8x0.8mm³), and a diffusion scan (identical). Following N4-bias correction, and T1-coregistration, two expert MRI technicians (>10 years of experience) manually segmented T2-hyperintense lesions using ITK-SNAP (www.itksnap.org) from FLAIR images. Lesion masks were used to in-fill MPRAGE scans using FSL lesion_filling."
Bias	9	Describe any efforts to address potential sources of bias	page 6 (methods)	"Along with the post-COVID patient group, we prospectively recruited a healthy control group without history of neurological or psychiatric diseases and without previous COVID-19 infection. Healthy control participants were individually matched regarding age, sex, and education (n=47, mean age 44·5 (14·1) years) and received an identical study protocol."
			page 19 (discussion)	"Given the overall impact of the pandemic restrictions on public health and well-being, it could be argued that changes in sleep and mood are expected around 1·5-2 years into a global pandemic. To account for this, we studied carefully matched, non-COVID and non-fatigue controls between April and November 2021 that had experienced the same pandemic restrictions as the post-COVID patient group, causing e.g., social isolation, job insecurity and unhealthy work-life balance patterns. Consequently, the here observed group differences of fatigue-related changes in mood, energy levels, and sleep are very likely attributable to the post-COVID syndrome."

Study size	10	Explain how the study size was arrived at	page 2 (abstract)<br><br>page 6 (methods)	"[…] moderate or severe fatigue was identified in 47/50 post-COVID patients who were included in the analyses."<br><br>"We prospectively recruited 50 patients from the neurological post-COVID outpatient clinic at Charité – Universitätsmedizin Berlin between April and November 2021. […]We identified 47 patients (mean age 43·4 ±11·9 years) with moderate or severe fatigue levels based on the cut-offs provided by the Fatigue Scale for Motor and Cognitive Function (FSMC ≥53) who were included in this cross-sectional study for further analyses."

Continued on next page

Quantitative variables	11	Explain how quantitative variables were handled in the analyses. If applicable, describe which groupings were chosen and why	page 7 (methods)	"Volumes were individually adjusted for a participant's whole brain volume (wBV) using the formula: volumeadjusted = volumeobserved - β [slope from wBV vs. regional volume regression] x (wBVobserved - wBVsample mean). We subsequently used these segmentations to create deformable surface meshes of the structures of interest using the vertex analysis implemented in FIRST. Surface deformations were compared between groups using FSL Randomise with TFCE and plotted using SurfIce (https://www.nitrc.org/projects/surfice/). Lastly, we used the segmentations of the individual structures to extract diffusion parameters from the preprocessed diffusion maps for each participant."
			page 7 (methods)	"Hypothesis-based analyses included volumetric and diffusion imaging analyses of thalamus, basal ganglia, and striatum. Our experimental hypothesis assumed that post-COVID fatigue was associated with structural imaging alterations of one or more subcortical structures. After visual inspection of boxplots to identify potential outliers, we used lme4 in R for a linear mixed effects analysis of group differences between patients and healthy controls by modelling the pairing of participants as random intercept. Confirmatory analyses were corrected for multiple comparisons using the Benjamini-Hochberg correction on the brain structure level. Moreover, exploratory analyses included diffusion imaging analyses of associated white matter tracts and correlational analyses with clinical and cognitive scores using Pearson or Spearman correlations, respectively."
Statistical methods	12	(a) Describe all statistical methods, including those used to control for confounding	page 8 (methods)	"Hypothesis-based analyses included volumetric and diffusion imaging analyses of thalamus, basal ganglia, and striatum. Our experimental hypothesis assumed that post-COVID fatigue was associated with structural imaging alterations of one or more subcortical structures. After visual inspection of boxplots to identify potential outliers, we used lme4 in R for a linear mixed effects analysis of group differences between patients and healthy controls by modelling the pairing of participants as random intercept. Confirmatory analyses were corrected for multiple comparisons using the Benjamini-Hochberg correction on the brain structure level. Moreover, exploratory analyses included diffusion imaging analyses of associated white matter tracts and correlational analyses with clinical and cognitive scores using Pearson or Spearman correlations, respectively."
		(b) Describe any methods used to examine subgroups and interactions	NA	NA
		(c) Explain how missing data were addressed	page 8 (methods)	"Missing data points, if applicable, were handled according to the recommendations provided in the test manuals."
		(d) Cohort study—If applicable, explain how loss to follow-up was addressed<br>Case-control study—If applicable, explain how matching of cases and controls was addressed<br>Cross-sectional study—If applicable, describe	page 6 (methods)	"Along with the post-COVID patient group, we prospectively recruited a healthy control group without history of neurological or psychiatric diseases and without previous COVID-19 infection. Healthy control participants were individually matched regarding age, sex, and education (n=47, mean age 44·5 (14·1) years) and received an identical study protocol."

		analytical methods taking account of sampling strategy		
		(e) Describe any sensitivity analyses	page 8 (methods)	"Lastly, we used a multiple linear regression analysis to identify potential predictors of post-COVID fatigue outcomes. Following the Shapiro-Wilk normality and log-transformation, if applicable, our model included clinical and neuropsychiatric predictors (as presented in fig. 4) and was controlled for multicollinearity. P-values represent 2-sided significance tests with an alpha level of α=0·05. Statistical analyses were performed using R 4·1·2."
**Results**				
Participants	13*	(a) Report numbers of individuals at each stage of study—eg numbers potentially eligible, examined for eligibility, confirmed eligible, included in the study, completing follow-up, and analysed	page 6 (methods)	"We prospectively recruited 50 patients from the neurological post-COVID outpatient clinic at Charité – Universitätsmedizin Berlin between April and November 2021. […] We identified 47 patients (mean age 43·4 ±11·9 years) with moderate or severe fatigue levels based on the cut-offs provided by the Fatigue Scale for Motor and Cognitive Function (FSMC ≥53) who were included in this cross-sectional study for further analyses."
			page 10 (results)	"Detailed clinical information about the acute COVID-19 disease stage is presented in table 1. Patients were predominantly female (39/47). The previous medical history in this patient sample included asthma (6/47, 13%), allergies/atopic dermatitis (6/47, 13%), hypertension (5/47, 11%), hypothyroidism (4/47, 9%), coagulation disorder (2/47, 4%), and breast cancer (1/47). Two patients reported infrequent migraine. In addition, three patients reported having had episodes of depression, anxiety and eating disorder throughout their lifetime. Since the onset of COVID-19, four patients had new-onset hypertension, two were diagnosed with asthma, and one patient developed heart problems, including arrhythmias and angina pectoris."
		(b) Give reasons for non-participation at each stage	NA	NA
		(c) Consider use of a flow diagram	NA	NA
Descriptive data	14*	(a) Give characteristics of study participants (eg demographic, clinical, social) and information on exposures and potential confounders	page 10 (results)	"Detailed clinical information about the acute COVID-19 disease stage is presented in table 1. Patients were predominantly female (39/47). The previous medical history in this patient sample included asthma (6/47, 13%), allergies/atopic dermatitis (6/47, 13%), hypertension (5/47, 11%), hypothyroidism (4/47, 9%), coagulation disorder (2/47, 4%), and breast cancer (1/47). Two patients reported infrequent migraine. In addition, three patients reported having had episodes of depression, anxiety and eating disorder throughout their lifetime. Since the onset of COVID-19, four patients had new-onset hypertension, two were diagnosed with asthma, and one patient developed heart problems, including arrhythmias and angina pectoris."<br>"At the time of the study visit, spontaneously reported complaints were feeling exhausted (39/47, 83%), difficulty concentrating (39/47, 83%), forgetfulness (29/47, 62%), feeling tired (26/47, 55%), word finding difficulties (21/47, 45%),

				headache (19/47, 40%), and feeling stressed/less resilient (17/47, 36%). In contrast, respiratory or systemic inflammatory symptoms were less common and included persistent dyspnoea (12/47, 26%), muscle or joint pain (9/47, 19%), chest pain (6/47, 13%), and flu-like symptoms (5/47, 11%) in some patients." "Both physical and cognitive fatigue were similarly affected in patients with post-COVID fatigue (table 1). Additionally, we observed increased levels of depressive symptoms (BDI-II: 17·0 (7·8) vs. 4·8 (5·4), b=12·1 [9·4, 14·9], p<0·001), anxiety (BAI: 14·6 (7·5) vs. 3·9 (4·8), b=10·7 [8·1, 13·3], p<0·001), daytime sleepiness (ESS: 11·2 (5·6) vs. 5·5 (3·2), b=5.6 [3.7, 7.5], p<0·001), and sleep problems (PSQI: 8·2 (3·9) vs. 5·8 (4·1), b=2.3 [0·7, 4·0], p=0·01) compared to healthy controls (fig. 1). The most affected PSQI subscales were daytime dysfunction (1·85 (0·79)), subjective sleep quality (1·54 (0·78)), and sleep disturbances (1·46 (0·60)). Moreover, short-term memory was impaired in patients compared to controls (RAVLT first recall: 6·1 (1·5) vs. 6·8 (1·9), b=-0·8 [-1·5, -0·04], p=0·04). On the test of higher attention, patients with post-COVID fatigue showed slowed response times (visual: 777 (83)ms vs. 710 (60)ms, b=67·3 [36·4, 98·1], p<0·0001; auditory: 628 (133)ms vs. 533 (83)ms, b=94·4 [49·1, 139·8], p<0·0001), while maintaining adequate accuracy (errors: 2·4 (1·8) vs. 1·9 (1·9), b=0·5 [-0·3, 1·2], p=0·21)."<br><br>Table 1. Clinical characteristics
		(b) Indicate number of participants with missing data for each variable of interest	NA	NA
		(c) Cohort study—Summarise follow-up time (eg, average and total amount)	NA	NA
Outcome data	15*	Cohort study—Report numbers of outcome events or summary measures over time	NA	NA
		Case-control study—Report numbers in each exposure category, or summary measures of exposure	NA	NA
		Cross-sectional study—Report numbers of outcome events or summary measures	Tables	Table 1. Clinical characteristics<br>Table S1. Structural integrity of subcortical structures.
Main results	16	(a) Give unadjusted estimates and, if applicable, confounder-adjusted estimates and their precision (eg, 95% confidence interval). Make clear which confounders were adjusted for and why they were included	page 7 (methods)	"Volume estimates of subcortical structures were obtained using model-based segmentation in FSL FIRST.20 The quality of automated segmentations was stringently visually controlled. Volumes were individually adjusted for a participant's whole brain volume (wBV) using the formula: volumeadjusted = volumeobserved - β [slope from wBV vs. regional volume regression] x (wBVobserved - wBVsample mean)."
		(b) Report category boundaries when continuous variables were categorized	pages 8 (methods)	"Specifically, the FSMC allows to distinguish between physical (e.g., effects of fatigue on strength, speed, or resting periods; 10 items) and cognitive symptoms (e.g., effect on concentration, memory, or attention, 10 items). Cut-offs exist for

				the total score classifying mild (≥43), moderate (≥53), and severe fatigue (≥63), as well as for the individual subscales.”
		(c) If relevant, consider translating estimates of relative risk into absolute risk for a meaningful time period	NA	NA

Continued on next page

Other analyses	17	Report other analyses done—eg analyses of subgroups and interactions, and sensitivity analyses	page 16 (results)	“In a multiple linear regression analysis, sleep quality (β=-0·48, p=0·01) and depressiveness (β=0·52, p=0·04) emerged as significant predictors of post-COVID fatigue (R^2=0·81, F(9,12)=5·62, p=0·01). In contrast, fatigue severity was unrelated to variables of the acute COVID-19 disease, including sex (female: 77·2 (10·4), male: 76·4 (9·4), t(9·3)=0·19, p=0·86), age at onset (r=-0·028, p=0·86), the duration (r=0·227, p=0·17) and number of symptoms during acute COVID-19 (r=-0·120, p=0·58) and the duration of post-COVID symptoms (r=0·030, p=0·85; fig. 4A).”
**Discussion**				
Key results	18	Summarise key results with reference to study objectives	page 2 (abstract)	“Our diffusion imaging analyses revealed aberrant fractional anisotropy of the thalamus. Diffusion markers correlated with fatigue severity, such as physical fatigue, fatigue-related impairment in everyday life (Bell score) and daytime sleepiness. Moreover, we observed shape deformations and decreased volumes of the left thalamus, putamen, and pallidum which overlapped with the more extensive subcortical changes in MS and were associated with impaired short-term memory. Post-COVID fatigue severity was not related to COVID-19 disease courses (6/47 hospitalised, 2/47 with ICU treatment). In contrast, post-acute sleep quality and depressiveness emerged as significant determinants and were accompanied by increased levels of anxiety and daytime sleepiness compared to controls.”
			page 18 (discussion)	“Our study shows that post-COVID fatigue is associated with distinct structural brain alterations in subcortical hubs that are detectable using MRI. Specifically, we identified reduced volumes and aberrant diffusion markers of the thalamus and basal ganglia that correlated with fatigue severity and impairment in daily activities, daytime sleepiness, and short-term memory problems. Importantly, this pattern of pathological changes emerged even though this cohort is relatively young, most patients were not hospitalised during their acute infection, and patients were in overall good health before COVID-19. Our novel finding - that post-COVID fatigue is associated with structural brain damage - highlights the importance of consequent therapeutic management of this debilitating postinfectious syndrome.”
			page 21 (discussion)	“In conclusion, our analyses show that a distinct pattern of thalamic and basal ganglia changes is associated with post-COVID fatigue. Imaging alterations

				include volume reductions, surface deformations, and aberrant diffusion markers that correlate with the severity and everyday impact of fatigue symptoms. Moreover, we show that post-COVID fatigue needs to be managed in a wider clinical array that also considers sleep quality, mood alterations, and cognitive impairment. Future research will determine whether these fatigue symptoms are transient or persistent. The identification of distinct subcortical brain correlates provides a foundation for further research on the pathomechanisms of post-COVID fatigue.”
Limitations	19	Discuss limitations of the study, taking into account sources of potential bias or imprecision. Discuss both direction and magnitude of any potential bias	page 20 (discussion)<br><br>page 20 (discussion)	“The high proportion of female patients in our study sample (39/47 patients) reflects the higher prevalence of fatigue in women that has also been reported in post-COVID syndrome.8,29 Although the mechanisms underlying these sex differences are not yet fully understood, a higher susceptibility to immune-mediated diseases may be a key factor.”<br><br>“Furthermore, it is currently unknown whether these post-COVID symptoms of fatigue and cognitive problems are transient or persistent. While we provide a comprehensive analysis of post-COVID fatigue, this study underlies the general limitations of cross-sectional designs. Due to the novelty of the disease, some patients in our cohort had COVID-19 less than six months ago. Future longitudinal designs are needed to determine the duration and course of the symptoms, and ultimately inform the prognosis of post-COVID syndrome.”
Interpretation	20	Give a cautious overall interpretation of results considering objectives, limitations, multiplicity of analyses, results from similar studies, and other relevant evidence	page 18 (discussion)<br><br>page 19 (discussion)<br><br>page 19 (discussion)<br><br>page 21 (discussion)	“Our novel finding - that post-COVID fatigue is associated with structural brain damage - highlights the importance of consequent therapeutic management of this debilitating postinfectious syndrome.”<br><br>“These findings suggest that a distinct pattern of pathological brain changes gives rise to fatigue symptoms across diseases, similar to previous descriptions of shared correlates of memory dysfunction in neuroimmunological disorders.19 The molecular pathophysiology underlying fatigue is, however, still unclear. Although studies reporting cytokine and endocrine abnormalities suggest an immune-mediated mechanism,26 there is currently no effective cell-based or serum marker available for clinical practice.”<br><br>“Given our current findings on basal ganglia damage and the accumulating evidence of a neuroimmunological aetiology,37 a disruption of cortico-striatal circuits together with dopaminergic imbalance in the basal ganglia might contribute to post-COVID fatigue.”<br><br>“Moreover, we show that post-COVID fatigue needs to be managed in a wider clinical array that also considers sleep quality, mood alterations, and cognitive impairment. Future research will determine whether these fatigue symptoms are

				transient or persistent. The identification of distinct subcortical brain correlates provides a foundation for further research on the pathomechanisms of post-COVID fatigue.”
Generalisability	21	Discuss the generalisability (external validity) of the study results	page 18 (discussion)	“Although fatigue is a subjective symptom based on self-report, 6 it has substantial and relevant consequences for a patient’s everyday life. The continuous experience of overwhelming exhaustion and low energy levels impacts quality of life,11 and around 30-50% of patients are unable to return to their previous workplace.12 Results on associated risk factors are heterogeneous, but accumulating evidence suggests that early neurological involvement may be an important precondition of long-term fatigue.13,29”
			page 19 (discussion)	“These findings suggest that a distinct pattern of pathological brain changes gives rise to fatigue symptoms across diseases, similar to previous descriptions of shared correlates of memory dysfunction in neuroimmunological disorders.24”
			page 20 (discussion)	“The high proportion of female patients in our study sample (39/47 patients) reflects the higher prevalence of fatigue in women that has also been reported in post-COVID syndrome.13,38 Although the mechanisms underlying these sex differences are not yet fully understood, a higher susceptibility to immune-mediated diseases may be a key factor.39”
			page 20 (discussion)	“While we provide a comprehensive analysis of post-COVID fatigue, this study underlies the general limitations of cross-sectional designs. Due to the novelty of the disease, some patients in our cohort had COVID-19 less than six months ago. Future longitudinal designs are needed to determine the duration and course of the symptoms, and ultimately inform the prognosis of post-COVID syndrome.”
**Other information**				
Funding	22	Give the source of funding and the role of the funders for the present study and, if applicable, for the original study on which the present article is based	page 9 (methods)	“Role of the funding source: The funding source had no role in study design, data collection, data analysis, data interpretation, or writing of the report.”

*Give information separately for cases and controls in case-control studies and, if applicable, for exposed and unexposed groups in cohort and cross-sectional studies.

Note: An Explanation and Elaboration article discusses each checklist item and gives methodological background and published examples of transparent reporting. The STROBE checklist is best used in conjunction with this article (freely available on the Web sites of PLoS Medicine at http://www.plosmedicine.org/, Annals of Internal Medicine at http://www.annals.org/, and Epidemiology at http://www.epidem.com/). Information on the STROBE Initiative is available at www.strobe-statement.org.
